# Antidepressant Effects of Essential Oils: A Review of the Past Decade (2012–2022) and Molecular Docking Study of Their Major Chemical Components

**DOI:** 10.3390/ijms24119244

**Published:** 2023-05-25

**Authors:** Emily Christie M. Fonseca, Lanalice R. Ferreira, Pablo Luis B. Figueiredo, Cristiane do Socorro F. Maia, William N. Setzer, Joyce Kelly R. Da Silva

**Affiliations:** 1 Programa de Pós-Graduação em Farmacologia e Bioquímica, Universidade Federal do Pará, Belém 66075-110, Brazil; emily.fonseca@icen.ufpa.br (E.C.M.F.); crismaia@ufpa.br (C.d.S.F.M.); 2 Programa de Pós-Graduação em Química, Universidade Federal do Pará, Belém 66075-110, Brazil; lanalice.ferreira@icen.ufpa.br; 3 Laboratório de Química dos Produtos Naturais, Centro de Ciências Biológicas e da Saúde, Universidade do Estado do Pará, Belém 66087-662, Brazil; pablo.figueiredo@uepa.br; 4 Aromatic Plant Research Center, Lehi, UT 84043, USA

**Keywords:** monoterpenes, sesquiterpenes, phenylpropanoids, linalool, β-caryophyllene, 5-HT receptor, serotonin receptor, monoaminergic, dopamine transporter, BDNF

## Abstract

Depression is a mental disorder that affects more than 300 million people worldwide. The medications available for treatment take a long time to exhibit therapeutic results and present several side effects. Furthermore, there is a decrease in the quality of life of people suffering from this affliction. Essential oils are traditionally used to relieve the symptoms of depression due to the properties of the constituents of these oils to cross the blood–brain barrier acting on depression-related biological receptors associated with reduced toxicity and side effects. In addition, compared to traditional drugs, they have several administration forms. This review provides a comprehensive assessment of studies on plants whose essential oil has exhibit antidepressant activity in the past decade and the mechanism of action of the major components and models tested. An additional in silico study was conducted with the frequent compounds in the composition of these essential oils, providing a molecular approach to the mechanism of action that has been reported in the past decade. This review is valuable for the development of potential antidepressant medications in addition to providing a molecular approach to the antidepressant mechanism of action of the major volatile compounds that have been reported in the past decade.

## 1. Introduction

Depression is a mental disorder representing a significant and growing public health problem, with an estimated 300 million people afflicted worldwide [1]. The COVID-19 pandemic increased the number of anxiety and depression disorders by 25% during its first year, and the latest data from the World Health Organization estimate that 71% of people with depression do not receive mental health services [2,3]. In addition to the problem of depression itself, this disease brings with it medium- and long-term consequences, such as cognitive disorders, which include deficits in several domains (attention, executive functions, memory and processing speed), dementia and is an initial cause of Parkinson’s disease [4,5].

Although drugs are available, access to pharmacological treatment for depression presents difficulties. The prescribed therapy is expensive, and only a small percentage of patients achieve remission with antidepressant monotherapy alone [6]. Another less discussed factor, a common problem affecting 30% to 50% of people with neurological diseases, is pharmacological refractoriness [7,8].

Refractory patients do not respond adequately to medications, even if administered correctly [9]. This reason is still not fully understood, but it is believed that some neurological diseases, because they are multifactorial, are involved in several biological aspects [10]. Thus, treatment may be effective for depression related to a specific etiological factor but not for depression secondary to another etiology [11].

The main classes of drugs available for the treatment of depression are selective serotonin reuptake inhibitors (SSRIs), serotonin noradrenaline reuptake inhibitors (SNRI), tricyclic antidepressants (TCAs) and monoamine oxidase inhibitors (MAOIs) [12]. The class used as the first choice for the treatment of depression is the SSRI, because, according to the pharmacological aspects, they are safe in overdose, have relative tolerability, have a generic form and have a broad spectrum of use compared to other classes [13]. Due to their lipophilic characteristics, SSRIs, especially fluoxetine, have a large volume of distribution between 14 and 100 L/kg, which indicates extensive tissue accumulation, mainly in the lung [14,15]. For this reason, SSRIs can increase systole and diastole time (QT interval), which increases the chances of fatal arrhythmia [16]. Currently, the prescription of other classes of antidepressants is very restricted due to high toxicity and potentially lethal food interactions [17]. For example, the Class of MAOIs interacts with foods containing tyrosine, increasing adrenergic activity at high risk of hypertensive crisis [18]. The class of TCAs also has effects on cardiac function such ashy postural hypo arrest and high-dose cardiotoxicity [19]. Prescription occurs only in patients who do not respond to more tolerable medications or have refractory depression [20]. In recent years, the antidepressant effect of ketamine anesthetic has been reported in cases of treatment-resistant depression [20]. However, ketamine in clinical practice was limited over a period of time due to its side effects on the central nervous system and the characteristics of a drug of abuse [21].

All classes of antidepressants, although with proven efficacy, have their limitations in terms of treatment. In general, the therapeutic effect appears within weeks of drug administration. On the other hand, side effects are felt first, directly interfering with the patient’s quality of life [22]. Among the main side effects, cardiac toxicity in case of overdose, dry mouth, accommodation disorders, constipation, urinary retention, sexual dysfunction and weight gain stand out [23,24,25]. In addition to these side effects, prolonged use of antidepressants can influence cognitive slowness and attention and memory difficulties. Despite being reversible, these effects lead some people to abandon therapy without completing treatment [26]. Given this context, it is important to identify new classes of antidepressant therapeutic agents with rapid onset of action, effective responses and fewer side effects.

The use of essential oils (EOs) is traditionally one of the complementary means or alternatives to drugs used in treating various diseases and symptoms, because they are a rich source of bioactive components [27]. The use of EOs has the advantage of topical or inhaled administration over orally administered drugs, which undergo first-pass metabolism, reducing the oral bioavailability and can provide drug interaction [28]. However, it is not because they are natural products that they can be used without worries. The main adverse events caused by the incorrect and indiscriminate use of EOs are dermatitis by allergic contact, phototoxicity and oral toxicity [29,30]. A review article published in 2022 on the toxicity of selected monoterpenes found that, while most are safe for human food and medical applications, there are monoterpene compounds that, in certain amounts or in particular circumstances (e.g., pregnancy), can cause serious disorders [31]. Therefore, the use of EOs should always be implemented with caution, because all substances can be toxic depending on the conditions of exposure, the dose and the route of administration [32].

In the past decade, many studies have reported the efficiency of EOs in relieving symptoms of diseases related to mental disorders, improving mood and feelings of physical and mental well-being, with the advantage of fewer adverse reactions [33]. The EOs from rose (*Rosa damascena* Mill.) [34], patchouli (*Pogostemon cablin* (Blanco) Benth.), lemongrass (*Cymbopogon citratus* (DC.) Stapf), sandalwood (*Santalum album* L.), bergamot (*Citrus bergamia* Risso & Poit.), valerian (*Valeriana officinalis* L.) and lemon (*Citrus limonum* Risso) are popularly consumed to relieve symptoms of depression and anxiety [29,35]. Thus, these popular uses stimulate investigations to understand the pharmacological processes of the antidepressant and anxiolytic effects of these EOs [36,37,38].

Lavender EO (*Lavandula angustifolia* Mill.) has been one of the most studied and has been shown to alleviate symptoms of anxiety and depression in clinical trials [39]. Lavender oil is rich in linalool and linalyl acetate, these two monoterpenoids being the components that determine the antidepressant effect [40]. The importance of natural products in the discovery of bioactive compounds has been extensively documented and has contributed to the development of current drugs [41,42]. For this reason, the identification of molecules in EOs with antidepressant action can contribute to primary or complementary therapy. In this context, in recent years, there has been an increasing number of publications on this topic [43]. However, the most recent review work published was in 2017, which carried out an EO survey of plants with antidepressant activity from 1995 to 2015 [44]. Therefore, this review provides a comprehensive update on EOs with antidepressant activity and aims to present the main results and antidepressant mechanisms obtained for the major constituents of these oils in silico, in vitro and in vivo models. In addition, this article developed an in silico approach to the mechanism of action of key compounds based on in vivo results, illustrating at the molecular level the interaction of these compounds at their corresponding target.

## 2. Molecular Mechanisms Involved in Depression

Depression is a complex and multifactorial illness triggered by psychological, genetic, social and biological factors [45]. Among the proposals on the biological etiology of depression, the classical theory suggests the hypothesis that the disease is due to a deficiency of monoamine neurotransmitters in the synaptic cleft, as noradrenaline (NA), dopamine (DA) and serotonin (5-HT) [46]. This proposition is reinforced by knowledge of the mechanism of action of antidepressants, including the drug Prozac^®^, which has the fluoxetine molecule as its active ingredient [47]. Currently, the evidence points to the neurochemical disturbance as a factor for depression disorder, which the 5-HT pathway is involved as one of the neurotransmission systems modified [48]. In addition, antidepressant use was linked to a reduction in 5-HT levels that was not associated to the depression state. Recently, in a systematic review, the authors infer the lack of a robust evidence of association between the reduced 5-HT levels or serotoninergic route hypoactivity per se and depression [48]. Thus, additional components may share the complex pathophysiological mechanisms, in which a cascade initiated in stressor condition on a susceptible genetic profile develops altered responses in an immunologic and endocrine scenario, eliciting functional and biochemical changes in central nervous regions by neurotrophic mediator alterations, neuroinflammation, oxidative stress and also neurochemical modifications [49].

In the past decade, most studies on the antidepressant action of EOs have been associated with more than one mechanism of action. However, the monoaminergic pathway involving the neurotransmitters DA and 5-HT is the most expressively elucidated, probably because of the monoamine theory concerning the most discussed pathway linked to depression. The second most discussed mechanism involves the participation of neurotrophic factors such as the Brain-Derived Neurotrophic Factor (BDNF). BDNF is related to synaptic plasticity and neurogenesis [50]. Thus, recent studies report that its decrease can cause different changes in the nervous system, such as depression, anxiety, schizophrenia and Parkinson’s disease [51]. In addition to the monoaminergic pathway and BDNF, other mechanisms that may be involved in depression have been reported, such as the GABAergic system alteration, increased expression of postsynaptic serotonin receptors (5-HT1A), decreased calcium influx and increased expression of astrocytes. The mechanisms of action of EOs with antidepressant effects reported in the past ten years are shown in Table 1.

## 3. Major Volatile Compounds of Essential Oils with Antidepressant Action

The EOs that showed antidepressant effects have two classes of major compounds in common: terpenoids and phenylpropanoids. The following subtopics describe some studies on the main antidepressant effects and mechanisms of these compounds.

### 3.1. Monoterpenes with Antidepressant Action

The EO samples rich in monoterpenes (19 samples) showed linalool, d-limonene, α-phellandrene, γ-terpinene and terpinen-4-ol, as the most frequent compounds. EOs of *Aniba rosiodora*, *Aeollanthus suaveolens* and *Aniba parviflora* rich in linalool (88.6%, 49.3% and 45.0%, respectively) were evaluated for the neurobehavioral effect in Wistar rats at doses of 3.5 and 35 mg/kg intraperitoneally (i.p.). Linalool was responsible for the significant improvement in symptoms of depression similar to fluoxetine (10 mg/kg) through its action on the serotonergic pathway [33].

Inhalation of EO from *Citrus sinensis* and *C. reticulata*, rich in d-limonene (90.7% and 76.7%, respectively), significantly improved depression-like behavior in mice, suggesting the involvement of the main compound in key mechanisms such as increased expression of BDNF and 5-HT1A receptor, which results in neurogenesis and serotoninergic pathway improvement, respectively [67,80]. *Origanum majorana* EO rich in terpinen-4-ol (32.6%) showed antidepressant effects in mice at doses of 10 and 80 mg/kg comparable to fluoxetine (20 mg/kg), used as a positive control. The results indicated the involvement of EOs in dopaminergic (D1 and D2), serotonergic (5HT1A, 5-HT2A receptors) and noradrenergic (α1 and α2 adrenoceptors) receptors. On the other hand, terpinen-4-ol is probably responsible for the antidepressant activity involved in the monoaminergic system [80].

### 3.2. Sesquiterpenes with Antidepressant Action

Despite the wide distribution of sesquiterpenes in essential oils, there are few studies on their antidepressant action [89]. *Anthriscus nemorosa* EO presented β-caryophyllene (23.6%), caryophyllene oxide (12.3%) and δ-cadinene (12.10%) as major components that were tested in mice by inhalation (1% to 3%). Results demonstrated antidepressant and an anxiolytic response attributed to β-caryophyllene, which positively modulated the GABA_A_ receptor activity similar to the positive control group treated with diazepam (1.5 mg/kg) [61].

EOs from *Pinus halepensis* having a β-caryophyllene content of 29.4% have been evaluated in vivo by inhalation (1% and 3%) attenuating anxious-depressive behaviors in the model of Alzheimer’s disease induced by Aβ1-42 in rodents [77]. Recent studies published between 2019 and 2022 report the therapeutic potential of β-caryophyllene as a reducer of pro-inflammatory mediators, improving the symptoms of neurological diseases characterized by inflammation and oxidative stress [90,91,92,93]. All these pieces together suggest that β-caryophyllene may exhibits antidepressant activity through more than one mechanism of action in the pathophysiology of depression.

The antidepressant potential of *Pogostemon cablin* EO was investigated from different fractions with different concentrations of patchoulol. The separation process was carried out by vacuum distillation in the following temperature ranges resulting in patchoulol concentrations of 42.8%, 49.3% and 60.6%. The fraction with the highest patchoulol content (60.66%) showed a better antidepressant effect in animal models, suggesting a monoaminergic mechanism with increased dopamine availability [36].

β-Elemene is the second most widely distributed sesquiterpene in EOs with antidepressant action and was identified in *Magnolia sieboldii* (22.1%) and *Toona ciliata* (24.9%) oils. For the EO of *M. siebold*, at concentrations 625, 1250 and 2500 μL/kg, the antidepressant effect was attributed to the sesquiterpenes β-elemene and germacrene D, which increased the expression of BDNF and 5-HT1A in the brain tissue of mice, in addition to stimulating the secretion of serotonin [85]. *T. ciliata* EO produced antidepressant effects at 20, 40 and 80 mg/kg concentrations in mice by increasing DA, NE, 5-HT and BDNF levels in the hippocampus in a dose-dependent manner [60].

### 3.3. Phenylpropanoids with Antidepressant Action

The phenylpropanoid (*E*)-cinnamaldehyde (87.3%), the main component of the EO of *Cinnamomum verum*, was responsible for the reduction of the depressive effect in mouse models at doses of 0.5, 1.0 and 2.0 mg/kg; however, its mechanism of action was not elucidated [65]. EOs from *Foeniculum vulgare* and *Pimpinella anisum* showed 82.1% and 88.4% (*E*)-anethole concentrations, respectively. *Foeniculum vulgare* oil showed an antidepressant effect in mice treated i.p. (100 to 400 mg/kg) through DA and 5-HT pathways in addition to antioxidant activity [82]. *Pimpinella anisum* oil (0.3 mg/kg) indicated that it could alter the effect of drugs that influence the nervous system. The intake of EO led to a significant increase in the analgesic effect of codeine. Motor impairment caused by midazolam was greater in the group treated with the EO. The diazepam application indicated the drug’s increased effect on motor activity. The pretreatment with EO caused a significant reduction in the pentobarbital-induced sleep time when compared to the control. The pretreatment diminished the decrease in the antidepressant effect of imipramine and fluoxetine with aniseed EO. However, the mechanism of action of *Pimpinella anisum* EO still needs to be defined. Thus, the evaluation of results gained in our study together with the previously published data indicate that use of aniseed EO can change the effect of drugs that act in central nervous system [53].

## 4. Molecular Docking

**Figure 1:**
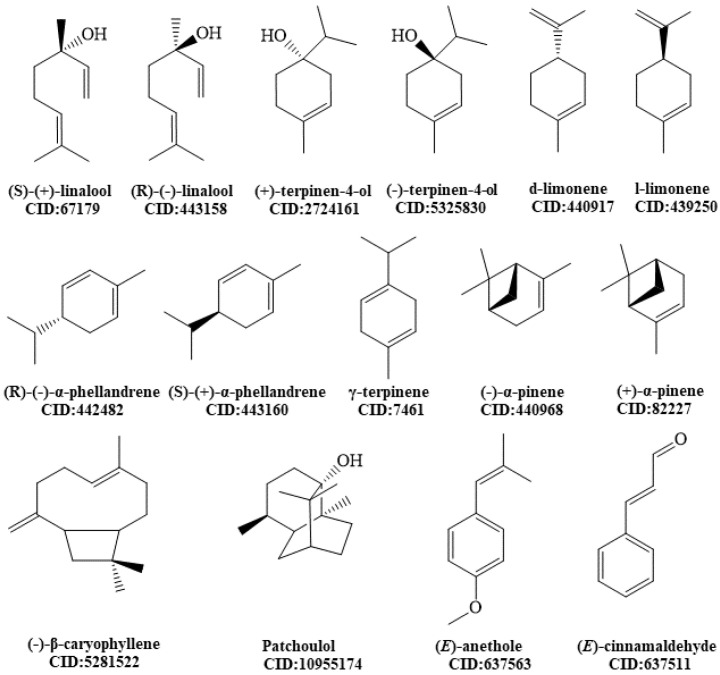
Chemical structures of molecules analyzed by molecular docking.

Molecular docking analysis was applied to elucidate the mechanisms of action of the compounds described in this study as antidepressants by in vivo models. The selected molecules (Figure 1) demonstrated a potential effect on the monoaminergic pathway, specifically on the serotonin transporter (SERT) and dopamine transporter (DAT).

Molecules were built and optimized using the Avogadro 1.2.0 software, as well as its main enantiomers, following the MMFF94 method to include all potential conformations in medium-sized rings and bonds, where interconversion between conformations may be impaired [94]. The 3D structures of SERT and DAT were obtained from the protein database (PDB) under code 5I6X and 4M48, respectively. The search sphere for each ligand was 12 Å in radius centered on the sites, and default protonation states of each protein based on neutral pH were used and charges were assigned based on default templates as part of the Molegro Virtual Docker program version 6.0 (MVD). The bonds of the compounds were taken as flexible, and the receptors were considered rigid. Different ligand orientations were generated and classified based on their energy scores. A minimum of 10 runs for each binder were performed. An energy score adjustment was also performed in order to eliminate the bias of the anchoring energies (E_dock_) with the increase of the second molecular weight, determined as DS_norm_, from the equation:

DS_norm_: 7.2 × E_dock_/MW^1/3.^ (1)

where DS_norm_ is the normalized docking score, E_dock_ is the Moldock reclassification score, MW is molecular weight and 7.2 is a scale constant to bring the average values of DS_norm_ comparable to E_dock_ [95]. The best-fitting results are summarized in Table 2.

Molecular docking was performed for all enantiomers to identify the possible enantioselectivity of these structures against the molecular target. Based on the affinity energy results, a significant difference is observed only from (*R*)-(−)-linalool at the DAT receptor, with affinity energy higher than (*S*)-(+)-linalool at the same receptor (−92.76 kJ/mol and −85.86 kJ/mol, respectively). There is evidence that the semantic treatment of linalool enantiomers separately promotes different effects once these compounds are chemically, biosynthetically, electrophysiologically and behaviorally distinct [96]. However, the elucidation of the relative stereochemistry of asymmetric centers of organic molecules is a challenge in the Natural products chemistry, because it requires the simultaneous determination of conformation and configuration, and few studies that discriminate the enantiomers and their mechanisms are related to depression [97].

**Figure 2:**
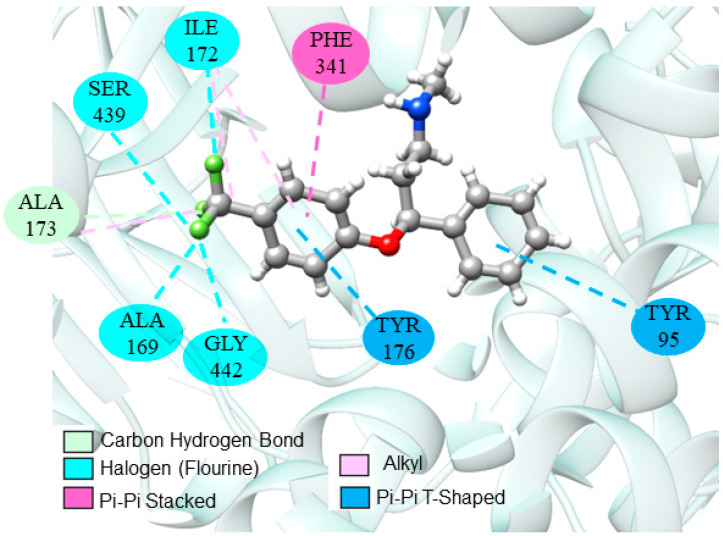
Interactions of fluoxetine at the SERT catalytic site.

Continuing the analysis, several studies reported the use of fluoxetine as a positive control in animal models. Interactions between fluoxetine and the SERT receptor are already well established and discussed in the literature [47,98,99]. Molecular interactions that configure the stability and substrate preference for the inhibitor are illustrated in Figure 2.

### 4.1. Monoterpenes

Monoterpenes show more exothermic docking results in the SERT receptor compared to the DAT receptor (Table 2). Specifically, d-limonene, γ-terpinene and α-phellandrene adopted an energy gain above 10 kJ/mol. The results show the compound preference for the SERT receptor, caused by the mechanism of serotonergic inclination, and corroborates the in vivo studies cited in this review.

The values of the affinity energy of monoterpenes are inferior compared to fluoxetine (antidepressant drug reference), which presented a value of −108.28 kJ/mol in the catalytic site. However, it is important to emphasize that it is a synthetic product and structurally differs from monoterpenes.

In addition, the docking energies of compounds are biased by larger molecular mass, since they have a greater number of atoms interacting with the target molecule. There will be a tendency for the selection of larger molecules, even if they are not necessarily as structurally complementary to the target binding site as the smaller compounds; adjustment of the docking score (DS_norm_) is required to correct this problem [100]. After that, we can compare the results of the indications of the EOs with the drug fluoxetine. (*S*)-(+)-linalool displayed an affinity energy value of −93.83 kcal/mol, very close to the value obtained for fluoxetine. The hydroxyl group in both oxygenated monoterpenes allow stronger interactions at the catalytic site and hydrophobic interactions promoting greater stability and affinity for the receptor.

**Figure 3:**
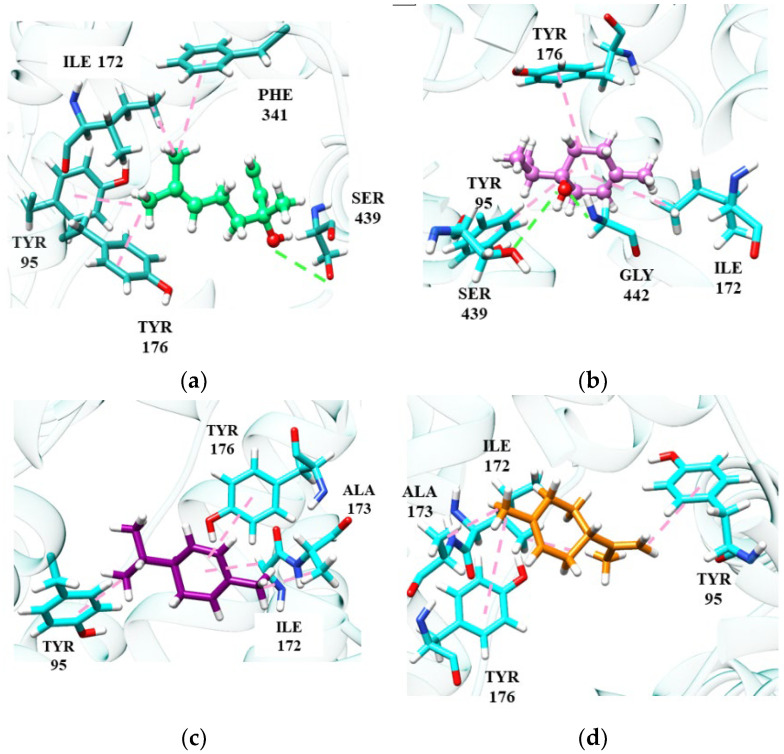
Monoterpene interactions at the SERT site: linalool (**a**), terpinen-4-ol (**b**), α-phellandrene (**c**), D-limonene (**d**).

Among the monoterpene hydrocarbons, we can highlight (*R*)-(−)-α-phellandrene and d-limonene, which showed the best affinity energies (−84.08 kJ/mol and −83.93 kJ/mol, respectively). In the absence of hydrogen bonds, hydrophobic interactions of the π-alkyl or π-pairing influence the stability of molecules at the catalytic site [101]. Despite being a weak interaction, hydrophobic interactions can play a significant role in the conformation and stability of structures and complexes, working cooperatively with energy values of –0.5 and −1.0 kJ/mol per interaction [102]. Figure 3 shows the main monoterpene interactions with the best binding energy results.

(*S*)-(+)-Linalool and (+)-terpinen-4-ol perform some important interactions in SERT inhibition, similar to the fluoxetine molecule. (*S*)-(+)-Linalool makes an π-alkyl interaction with residues Phe341, Tyr95 and Ile172, a significant interaction as it reproduces the docking effect of fluoxetine, as demonstrated in previous studies [103]. (*S*)-(+)-Linalool also showed relevant interactions with the residues of Ile172, Phe341 and Tyr95. A previous study demonstrated that the mutation in these residues configures a decrease of >10 in the Ki value of fluoxetine, suggesting that these are determinant residues for SERT inhibition [47]. (+)-Terpinen-4-ol replaced the interaction of Phe341 with Tyr176, which may be related to lower energy than that of (*S*)-(+)-linalool. Specifically, (+)-terpinen-4-ol made an additional energetically favorable hydrogen-bond interaction with the Gly442 residue. The hydrogen-bonding interaction of fluoxetine with Gly442 has previously been demonstrated [98]. The two hydrocarbon monoterpenes, (*R*)-(−)-α-phellandrene and d-limonene, also do not interact with Phe341 and have lower affinity energy than linalool and (+)-terpinen-4-ol. However, they still manage to interact with essential residues at the catalytic site.

### 4.2. Sesquiterpenes

Among the sesquiterpenes, we highlight (−)-β-caryophyllene and patchoulol, which are involved in activity against depression, however, acting on different targets (SERT and DAT, respectively). Patchoulol did not show good affinity energy (−56.12 kJ/mol) at the SERT receptor, suggesting a non-compatibility between the target and molecule. Differently, at the DAT receptor, patchoulol presented a better affinity energy (−72.52 kJ/mol), corroborating with the studies that identified the antidepressant activity of patchoulol in the dopaminergic pathway [36,104].

**Figure 4:**
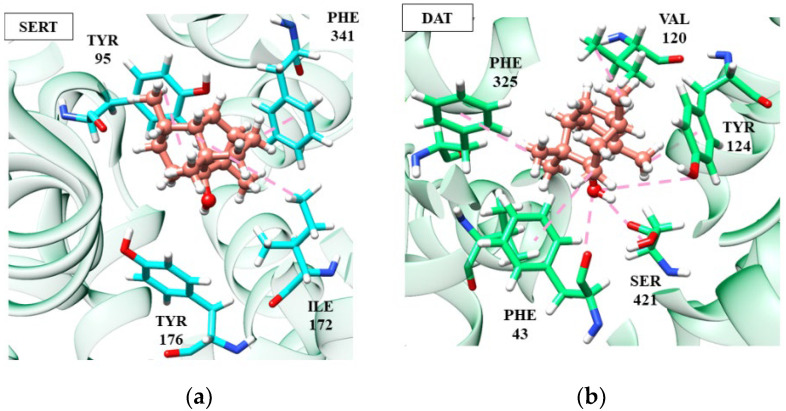
Patchoulol (salmon) interactions in SERT (**a**) and DAT receptors (**b**).

According to Figure 4, it is possible to notice that patchoulol fits better in the catalytic site of the DAT protein, performing interactions with a greater number of amino acid residues, which directly influences the affinity energy. In the patchoulol-DAT complex, hydrogen-bonding interactions occur between the hydroxyl group of the ligand and Ser421 and Tyr124 residues. In contrast, in the SERT protein, patchoulol does not interact strongly due to the 4.99 Å distance to the Tyr176 residue. Consequently, the dipole-induced interaction between Ile172 and Tyr176 is weakened, compromising energy redistribution in the site’s surroundings [69]. On the other hand, (−)-β-caryophyllene has good affinity at the SERT receptor (−96.20 kJ/mol) but has a significant loss in affinity towards the DAT receptor (−58.55 kJ/mol), suggesting greater performance in the serotonergic pathway [36,104].

**Figure 5:**
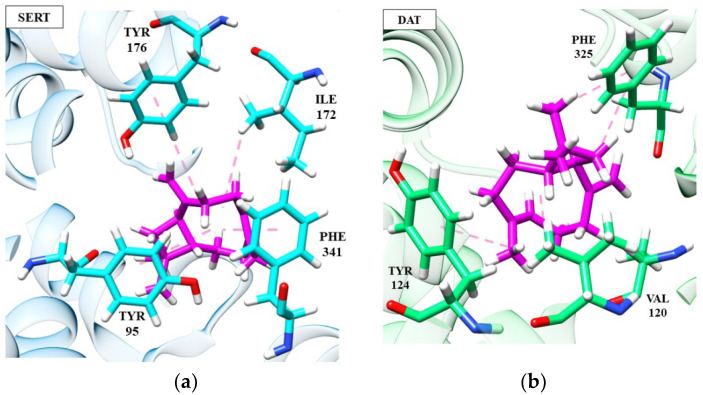
Interactions of (-)-β-caryophyllene (magenta) at SERT (**a**) and DAT (**b**) receptors.

According to Figure 4, it is possible to notice that patchoulol fits better in the catalytic site of the DAT protein, performing interactions with a greater number of amino acid residues, which directly influences the affinity energy. In the patchoulol-DAT complex, hydrogen-bonding interactions occur between the hydroxyl group of the ligand and Ser421 and Tyr124 residues. In contrast, in the SERT protein, patchoulol does not interact strongly due to the 4.99 Å distance to the Tyr176 residue. Consequently, the dipole-induced interaction between Ile172 and Tyr176 is weakened, compromising energy redistribution in the site’s surroundings [103]. On the other hand, (−)-β-caryophyllene has good affinity at the SERT receptor (−96.20 kJ/mol) but has a significant loss in affinity towards the DAT receptor (−58.55 kJ/mol), suggesting greater performance in the serotonergic pathway (Figure 5).

The (−)-β-caryophyllene molecule performs hydrophobic and electrostatic interactions with the SERT residues of Tyr176, Tyr95, Phe341 and Ile172, which are important for blocking serotonin transport, as discussed in previous topics. These interactions, except Tyr95, are absent in patchoulol, compromising its target affinity and indicating its relevance to the stability of the complexes. In the DAT receptor, both (−)-β-caryophyllene and patchoulol interact with the Val120 residue, which is largely conserved and faces the cycloheptene ring of Phe325. This triad of interactions is necessary for DAT blockade [105]. However, we can suggest that the lower affinity of (−)-β-caryophyllene at the DAT receptor is due to the absence of interaction between Ser421 and Phe43, which, on the other hand, is visualized in patchoulol.

Ser421 coordinates the sodium ion (cofactor). It makes a hydrogen interaction with the carbonyl of the Phe43 residue, and then these two residues participate in a network of hydrogen bonds that interconnect patchoulol. Such interactions have already been described as essential for ligand recognition and affinity [103]. The results obtained in this molecular docking analysis correlate with the antidepressant effects observed for the EOs of the species Anthriscus nemorosa and Pogostemon cablin [61].

### 4.3. Phenylpropanoids

**Figure 6:**
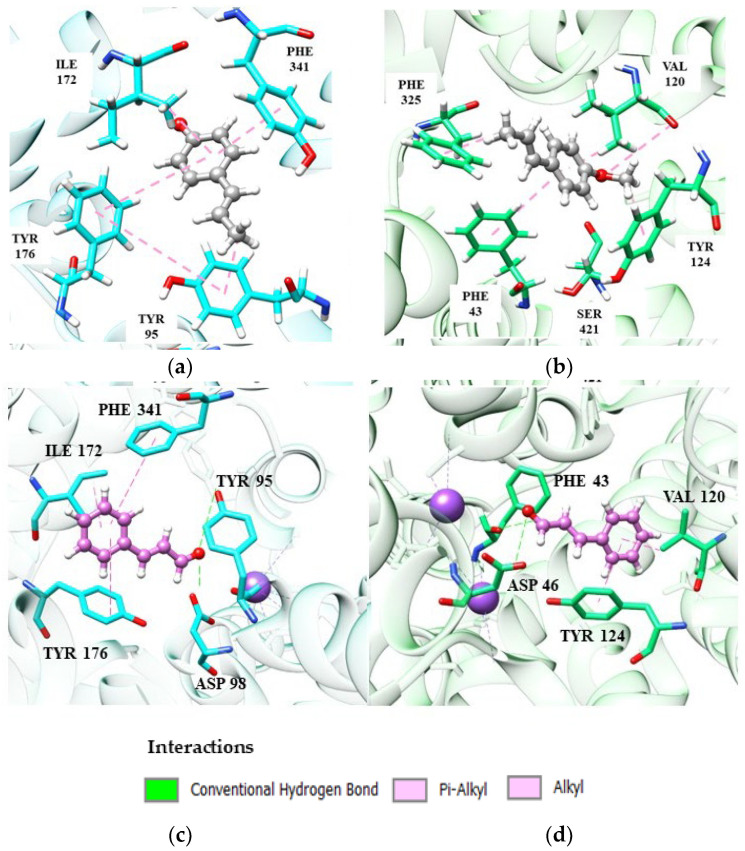
Interactions of (*E*)-anethole and (*E*)-cinnamaldehyde phenylpropanoids at the SERT and DAT site: (**a**,**b**) catalytic site of SER and DAT, respectively, complexed with (*E*)-anethole (dark gray); (**c**,**d**) catalytic site of SERT and DAT, respectively, complexed with (*E*)-cinnamaldehyde (magenta).

The compound (*E*)-anethole showed antidepressant activity in vivo by the monoaminergic mechanism, specifically 5-HT and DA. (*E*)-cinnamaldehyde showed an antidepressant effect without elucidation of the mechanism of action. For this reason, molecular docking of these two phenylpropanoids was carried out against the SERT and DAT receptors. Based on the docking energy result, (*E*)-anethole showed a higher affinity in the SERT (−87.82 kJ/mol) compared to the DAT (−64.05 kJ/mol), suggesting that the action in the dopaminergic pathway may not only be due to the blockade of the DAT. In contrast, (*E*)-cinnamaldehyde had similar affinity energies on the two targets with a slight preference for SERT. Figure 6 shows the interactions of the four complexes formed.

(*E*)-Anethole coordinates π-alkyl-like interactions between Ile172 and Tyr95 and π-pairing between Tyr176 and Phe341 with the receptor SERT (Figure 6). These interactions have been discussed in previous topics and are crucial for the binding and stability of a potential SERT inhibitor. (*E*)-Cinnamaldehyde (Figure 6C) also repeats the same interactions.

Evaluating the interactions present in DAT, the conformation adopted by the more stable (*E*)-anethole prevents the interaction between its carbonyl group with Ser421, negatively implying the affinity of the complex. (*E*)-Cinnamaldehyde also does not interact with Ser421, but its stability is not as compromised due to its conformation, which facilitates an additional hydrogen-bonding interaction between Phe43 and Asp46.

## 5. Molecular Docking (In Silico) Study: Final Considerations

Among the compounds evaluated in this study by molecular docking analysis, we can highlight those with better affinity energies against the two targets, specifically, (*S*)-(+)-linalool (SERT: −92.83 and DAT: −85.86), (*E*)-cinnamaldehyde (SERT: −86.56 kJ/mol and DAT: −72.76 kJ/mol) and γ-terpinene (SERT: −81.86 kJ/mol and DAT: −70.18). Most published studies describe the antidepressant effect of essential oils rich in monoterpenes. However, the results obtained in our in silico evaluation show that this property is not restricted to this class of compounds. For example, the sesquiterpene patchoulol demonstrated the best affinity results on the dopamine target. The phenylpropanoid (*E*)-anethole showed an affinity energy at the SERT receptor (−87.82 kJ/mol) similar to the monoterpenoid linalool.

The results obtained in the present study reinforce the importance of the synergistic effect between the components of the EOs, which may be involved in different mechanisms related to the pathophysiology of depression. Therefore, the therapeutic effect may not be directly linked to the concentration of a single component since different compounds can express favorable results, such as enhancing the effectiveness of the effect, minimizing or delaying the development of resistance and providing selective synergism against the target [106]. The in silico analysis was limited to evaluating only two targets of the monoaminergic pathway. However, depression, being a multifactorial disease, is far from being entirely explained by monoamine deficiency alone [107]. In this way, for the first time, this review work confirms, at the molecular level, some of the main mechanisms involved in depression described in the past ten years. More robust in silico techniques are needed to fully clarify the mechanism of antidepressant action of the compounds present in EOs. However, our results are in alignment with the in vivo results and demonstrate the binding energy influences of molecules against different targets.

## 6. Materials and Methods

**Figure 7:**
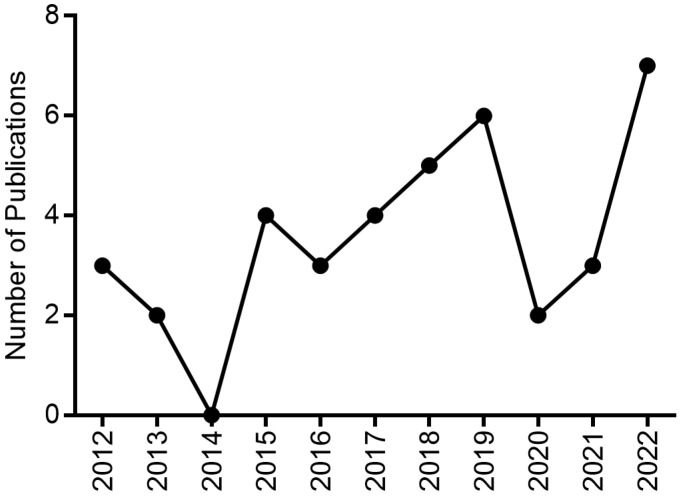
Graphical representation of the number of publications on EOs with antidepressant activity in the past decade.

The present study was based on scientific publications on EOs from plants with antidepressant activity between the years 2012 and 2022. Figure 7 emphasizes the gradual growth that publications on this topic were having from 2016 to 2019. The year 2020 is marked by the COVID-19 pandemic and the whole world joined forces in the search for measures against this disease [108,109]. In 2021 and 2022, there is an increase in publications on the topic of depression. For this reason, the production of this review highlighted aromatic plants with antidepressant activity, as well as the major chemical components of EOs, applied assay models (in silico, in vitro and in vivo) and their corresponding mechanisms of action, which are listed in Table 1.

### 6.1. Search Strategy and Inclusion and Exclusion Criteria

**Figure 8:**
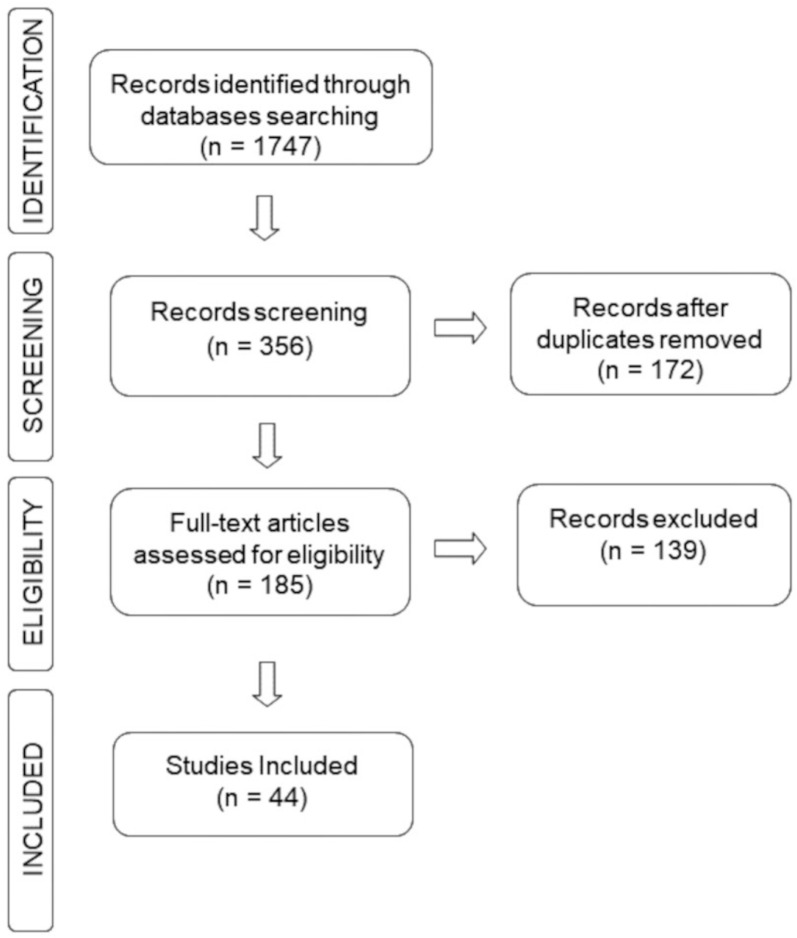
Search strategy flowchart.

The search for information on the chemical composition of EOs and tests performed were implemented considering all articles published in the past ten years (2012–2022) in the literature databases: Scopus (https://www.scopus.com, accessed on 17 November 2022), Science Direct (https://www.sciencedirect.com/, accessed on 18 November 2022) and PubMed (https://pubmed.ncbi.nlm.nih.gov/, accessed on 19 November 2022). The primary keywords “essential oil” or “volatile compounds” and “antidepressant effect” or “antidepressant activity” were searched and combined in the titles and abstract. Inclusion criteria for sections of this study were accessed for chemical composition and assays to support antidepressant activity. Searches performed with commercial samples of EO, review articles, books, book chapters and abstracts were excluded. Figure 8 summarizes the general methodology by highlighting the articles collected in each database, duplicate and deleted files and finally how many were selected for the writing of this review.

### 6.2. Study Records: Data Management

The mechanism used for data management is described in Table 1, informing the name of the plant species and main components of the EO, as well as the type of test carried out to evaluate the antidepressant effects of the EO. Two different reviewers completed the reading and revision of the cited articles in each selection phase.

## 7. Conclusions

EOs have been widely studied for their therapeutic properties and potential health benefits and as an alternative for the treatment of depression [44,108]. In this context, the search for bioactive molecules of natural origin is a promising area in pharmaceutical research, since many plants have compounds with proven antidepressant activity [110,111]. The research of bioactive compounds against depression of natural origin is important not only for the possibility of developing new safer and more effective drugs, but also for the valorization of traditional knowledge and biodiversity [112]. Many plant species that contain EOs with antidepressant activity widely studied, have shown promise for more advanced studies, including Roman chamomile (*Chamaemelum nobile* L.), rosemary (*Rosmarinus officinalis* L.) and sweet orange (*Citrus sinensis* (L.) Osbeck). These plants are good candidates for clinical trials due to their proven effects in reducing symptoms of depression, as well as being of low toxicity and being widely available and used in the cosmetics and food industry [113,114,115]. However, each plant species has a unique chemical composition, and the concentration and activity of bioactive components may vary in different cultivars or geographical origins [116]. For this reason, careful selection of the raw material used in the production of essential oils is fundamental to ensure the quality and effectiveness of the results of clinical studies [117]. Although EOs are often considered safe and natural, it is important to recognize that they are highly concentrated chemical compounds and should be used with caution [118]. Some EOs can cause skin or eye irritation and can be toxic if ingested in large quantities [119,120]. The specific side effects depend on the EO in question, as well as the dose and method of administration [121]. 

Therefore, this review offers an overview of the evidence found in the past ten years of the use of EOs with antidepressant activity as well as different routes of administration and the main mechanisms. In the general analysis performed, aromatherapy showed its potential to be used as an effective therapeutic option for the relief of depressive symptoms.

## Figures and Tables

**Table 1:** Summary of the main information and results found in the research of plant species that showed antidepressant activity in the past ten years.

Publication Year/Species	Major Compounds	Assays	RA	DR	PE or AM	Ref.
2012 *Lavandula terpinenetia* ssp. *terpinenenia* Mill.	linalool (28.0%); linalyl acetate (17.0%); terpinene-4-ol (3.3%)	YMT; EPM; FST; RAMT	INH	SLM (0.7 mg/kg) Silexan (200 µL)	Reduction of depressive- and anxiety-like behavior in models of dementia (Wistar rats, EO at 1% or 3%, 200 μL. Sub-chronic treatment).	[52]
2012 *Pimpinella anisum* L.	(*E*)-anethole (88.4%); γ-himachalene (3.1%); (*Z*)-isoeugenol (1.9%)	Hot-plate test; EPM; pentobarbital-induced sleeping time; FST; TST	PO	DZP (1.5 mg/kg) IPM (16 mg/kg) FLU (16 mg/kg)	Not reported (NMRI mice, EO at 0.3 mL/kg. Sub-chronic treatment).	[53]
2012 *Litsea glaucescens* Kunth	1,8-cineole (26.0%); *o*-cymene (25.9%); limonene (8.6%)	FST; OFT; exploratory cylinder; EPM; rotarod and traction performance	IP	IPM (30 mg/kg)	Antidepressant effect observed in the FST test (ICR mice, EO at 0.1 mL/10 g).	[54]
2013 *Caryophyllus aromaticus* L.	*bis*-eugenol (98% purity)	FST; TST; OFT	IP	DZP (2 mg/kg)	Antidepressant effect noted by the decrease in immobility time in the FST and TST mediated by the interaction with serotonergic, noradrenergic and dopaminergic (Swiss mice, EO at 10, 25 and 50 mg/kg. Acute treatment).	[55]
2013 *Rosmarinus officinalis* L.	1,8-cineole (45.1%); camphor (21.8%); α-pinene (4.7%)	TST; OFT	PO	FLU (10 mg/kg)	Antidepressant effect observed by reduction of immobility time in the TST similar to the positive control FLU (Swiss mice, EO at 0.1, 1, 10 and 100 mg/kg. Acute treatment).	[56]
2015 *Ferulago angulata* L.	α-pinene (24.10%); β-pinene (22.70%); β-phellandrene (20.50%)	FST; EPM/in silico: molecular docking	INH	DZP (1.5 mg/kg)	Inhibition of depressive behavior and reduction of anxiety in models of dementia Wistar rats (200 mL, 1% or 3%. Sub-chronic treatment).	[57]
2015 *Ocimum basilicum* L.	linalool (31.0%); estragole (15.5%); 1,8-cineole (3.2%)	EPM; FST	INH	DZP (1.5 mg/kg)	Depression parameters reduction in CUMS-induced models; decreased levels of corticosterone; increase in enzymatic antioxidants levels (Wistar rats, EO at 1% or 3%, 200 µL. Sub-chronic treatment).	[58]
2015 *Ocimum sanctum* L.	linalool (19.0%); estragole (7.5%); 1,8-cineole (3.9%)	EPM; FST	INH	DZP (1.5 mg/kg)	Depression parameters reduction in CUMS-induced models; decreased levels of corticosterone; increase in enzymatic antioxidants levels (Wistar rats, EO at 1% or 3%, 200 µL. Sub-chronic treatment).	[58]
2015 *Syzygium aromaticum* (L.) Merr. & L.M. Perry	eugenol (71.0%); 2-methoxy-4-isopropenylphenyl acetate (16.0%); β-caryophyllene (10.7%)	TST; FST; OFT; SPT; NSFT; tissue sample collection; Western blotting analyses	PO	IPM (15 mg/kg)	Antidepressant effect observed in behavioral tests; increased levels of ERK, p-CREB and BDNF (ICR mice, EO at 50, 100 and 200 mg/kg. Acute treatment).	[59]
2015 *Toona ciliata* M. Roem.	β-elemene (24.9%); β-cubebene (14.2%); estragole (6.1%)	FST; TST; OFT; SPT	PO	IPM (10, 20, 40 and 80 mg/kg)	Reduction of CUMS-induced serum corticosterone levels in rats; increase of neurotransmitters and BDNF (ICR mice, EO at 10, 20, 40 and 80 mg/kg. Acute treatment).	[60]
2016 *Anthriscus nemorosa* (M. Bieb.) Spreng.	β-caryophyllene (23.6%); caryophyllene oxide (12.3%); δ-cadinene (12.1%)	YMT; RAMT; EPM; FST	INH	DZP (1.5 mg/kg)	Memory enhancement with decrease in depressive-like behavior (Wistar rats, EO at 1% and 3%. Acute treatment).	[61]
2016 *Foeniculum vulgare* Mill.	camphor (21.3%); (+)-carvone (7.8%); *trans*-sabinene hydrate (3.1%)	EPM; FST	INH	DZP (1.5 mg/kg)	Antidepressant effect observed by increasing swimming time in a dose-dependent manner; decrease in immobility time in a dose-dependent manner (Wistar rats, EO at 1 or 3%. Sub-chronic treatment).	[62]
2016 *Pimpinella peregrina* L.	*trans*-pinocarveol (35.1%); pregeijerene (15.1%); α-cubebene (12.4%)	YMT; RAMT; EPM; FST	INH	DZP (1.5 mg/kg)	Increased memory; reduction of anxiety and depression in scopolamine-induced models (Wistar rats, EO 200 μL).	[63]
2017 *Chamaemelum nobile* (L.) Tudo.	α-pinene (% not reported)	OFT; FST	INH	Not reported	Increased the expression of proteins that are involved in the oxidative phosphorylation (Cytochrome c oxidase subunit 6C-2 and 7A2) (Wistar rats, EO 400 μL. Sub-chronic treatment).	[64]
2017 *Cinnamomum verum* J. Presl	(*E*)-cinnamaldehyde (87.3%)	FST; TST; EPM	IP	DZP (2 mg/kg) DPM (10 mg/kg) FLU (10 mg/kg)	Shorter duration of immobility time in the FST and TST tests (mice, EO at 0.5, 1 and 2 mg/kg. Acute treatment and Sub-acute treatment).	[65]
2017 *Ocimum basilicum* L.	linalool (35.9%); 1,8-cineole (11.2%); α-cadinol (10.4%)	FST; EPM; OFT	INH	FLU (20 mg/kg)	Antidepressant effect by decrease in corticosterone levels and increase in BDNF index; reduction of CUMUS-induced neuron atrophy; increased number of astrocytes comparable to fluoxetine (Swiss mice, EO at 2,5 mL. Sub-chronic treatment).	[66]
2017 *Ocimum basilicum* L.	linalool (35.9%); 1,8-cineole (11.2%); α-cadinol (10.4%)	CUMS; FST; EMP; OFT	INH	FLU (20 mg/kg)	Reduction of the depressive state evidenced in behavioral tests and antioxidant analysis (Swiss mice. Sub-chronic treatment).	[67]
2018 *Achillea biebersteinii* Afan.	1,8-cineole (33.2%); camphor (11.4%); 2-isopropyl-5-methyl-3-cyclohexen-1-one (10.4%)	YMT; RAMT; EPM; FST	INH	DZP (1 mL/kg)	Improved spatial memory, anxiety and reduction depressive-like behavior compared to rats treated with scopolamine alone (Wistar rats, EO at 200 μL, 1% or 3%. Sub-chronic treatment).	[68]
2018 *Aeollanthus suaveolens* Mart. ex Spreng	linalool (49.3%); (*E*)-β-farnesene (34.9%)	OFT; EPM; FST	IP	FLU (10 mg/kg)	Antidepressant effect is suggested due to the decrease in immobility time; increased self-cleaning time and interaction with the serotonergic system (Wistar rats, EO at 7.5 and 75 mg/kg. Acute treatment).	[69]
2018 *Aniba rosiodora* Ducke	linalool (88.6%)	OFT; EPM; FST	IP	FLU (10 mg/kg)	Antidepressant effect is suggested due to the decrease in immobility time; increased self-cleaning time and interaction with the serotonergic system (Wistar rats, EO at 7.5 and 75 mg/kg. Acute treatment).	[69]
2018 *Aniba parviflora* (Meisn.) Mez	linalool (45.0%); β-phellandrene (17.3%); α-phellandrene (4.1%)	OFT; EPM; FST	IP	FLU (10 mg/kg)	Antidepressant effect is suggested due to the decrease in immobility time; increased self-cleaning time and interaction with the serotonergic system (Wistar rats, EO at 7.5 and 75 mg/kg. Acute treatment).	[69]
2018 *Lippia sidoides* Cham.	thymol (79.7%)	FST; TST; EPMT; SPT	PO	FLU (35 mg/kg)	Increase in FST immobility time in corticosterone-induced rats (Swiss mice, EO at 100 and 200 mg/kg. Sub-chronic treatment).	[70]
2018 *Origanum vulgare* L.	thymol (20.7%); γ-terpinene (8.8%); borneol (8.7%)	SPT; FST	IP	DZP (3 mg/kg) FLU (20 mg/kg)	Depressive parameters reduction in models induced with CUMS. (Wistar rats, EO at 0.2 mL/kg. Chronic treatment).	[71]
2018 *Pelargonium roseum* Willd.	citronellol (35.9%); geraniol (18.5%); δ-selinene (5.5%)	EPM; OFT; FST	IP	DZP (1 mg/kg) BPR (10 mg/kg)	Antidepressant effect related to positive modulation of serotoninergic pathway, verified by the blockade of 5-HT1A receptor (Swiss mice, EO at 10, 20 and 50 mg/kg. Acute treatment).	[72]
2019 *Annona vepretorum* Mart.	(*E*)-β-ocimene (38.0%); spathulenol (14.0%); α-phellandrene (11.5%)	EPM; OFT	IP	FLU (20 mg/kg)	Decreased depressive behavior and regulation of 5-HT metabolism (Swiss mice, EO at 25, 50 and 100 mg/kg. Acute treatment).	[73]
2019 *Citrus sinensis* (L.) Osbeck	d-limonene (90.7%); α-pinene (5.9%); γ-terpinene (2.4%)	SPT; OFT; FST	INH	FLU (40 mg/kg)	Depression behavior reduction in mice induced by CUMS (EO 1 mL. Sub-chronic treatment).	[74]
2019 *Citrus sinensis* (L.) Osbeck	d-limonene (119.6 g/L); carveol (3.7 g/L); limonene oxide (2.5 g/L)	OFT	PO	RSP (1.0 mg/kg)	Reduction of depression-like behaviors by increased levels of 5-HT and DA in rat brains (mice, EO at 0.5%, 1% and 2%. Sub-chronic treatment).	[75]
2019 *Lavandula angustifolia* Mill.	linalyl acetate (35.9%) linalool (35.2%) β-caryophyllene (3.3%)	TST	PO	Citalopram (10 mg/kg)	LEO reduces symptoms of NIS-induced neuropathic pain. In rats, they simultaneously elicit an antidepressant and anxiolytic activity (CD1 mice, EO at 100 mg/Kg. Sub-chronic treatment).	[76]
2019 *Pinus halepensis* Mill.	β-caryophyllene (29.4%); α-pinene (11.1%); myrcene (7.8%)	EPM; FST; protein extraction; CAT, SOD and GPx assessment;	ICV, IP; INH	Donepezil (5 mg/kg)	Antidepressant effect by protection against neuroinflammation and neuroapoptosis (Wistar rats, EO at 1% or 3%, 200 μL). Acute treatment).	[77]
2019 *Pogostemon cablin* Benth	patchoulol (32.8%)	N/A	PO	Not reported	Reduction of depressive behavior by 76.33% in the group that received microcapsule powder of 1 g of maltodextrin, 1 g of gum arabic and 3 g of patchoulol (mice *Mus musculus*).	[78]
2020 *Aegle marmelos* (L.) Corrêa	d-limonene (46.0%); α-phellandrene (35.9%); α-pinene (5.6%)	EPM; HBT; TST */* in silico: molecular docking	IP	DZP (2 and 5 mg/kg); FLU (20 mg/kg)	Positive Modulation of the GABAergic and serotoninergic systems (Swiss mice, EO at 25–100 mg/kg. Acute treatment).	[79]
2020 *Origanum majorana* L.	terpinen-4-ol (32.6%); γ-terpinene (12.8%); *trans*-sabinene hydrate (8.4%)	FST; OFT	IP	FLU (5 and 20 mg/kg)	Antidepressant effect due to the involvement of the serotoninergic pathway, confirmed by the action of the antagonist on 5-HT1A receptors (Mice, EO at 10 and 80 mg/kg. Acute treatment).	[80]
2021 *Cinnamomum osmophloeum* Kaneh.	linalool (93.2%); coumarin (2.3%); camphor (1.5%)	OFT; FST; tail flick test; primidone-induced sleeping test; rotarod test;	PO	Trazodone (100 mg/kg)	Antidepressant effect evidenced by the decrease in immobility time in the FST (ICR mice, EO at 100, 200 and 400 mg/kg. Sub-chronic treatment).	[81]
2021 *Foeniculum vulgare* Mill.	(*E*)-anethole (82.1%); α-thujone (12.1%)	FST; OFT	IP	FLU (20 mg/kg)	Reduction of the depressive effect by the antioxidant action; reduction of immobility time with results similar to fluoxetine (mice, EO at 50–400 mg/kg. Acute treatment).	[82]
2021 *Piper nigrum* L.	limonene (25.3%); sabinene (22.8%); β-caryophyllene (13.3%)	TST; OFT	PO	DZP (1 mg/kg) BPR (10 mg/kg)	Antidepressant effect related to serotoninergic pathway verified by the blockade of 5-HT1A receptor (Swiss mice, EO at 5, 10 and 15 mg/kg. Sub-chronic treatment).	[83]
2022 *Citrus reticulata* Blanco	d-limonene (76.7%); β-myrcene (4.7%); γ-terpinene (4.4%)	FST; TST	INH	FLU (20 mg/kg)	Reduction of depression-like behaviors in reserpine-induced model depressed mice (mice, EO at 0.625, 1.25 and 2.5 mL/kg. Acute treatment).	[84]
2022 *Magnolia sieboldii K.* Koch	β-elemene (22.1%); (*E*)-β-ocimene (14.9%); germacrene D (7.3%)	FST; TST	IP	FLU (20 mg/kg)	Antidepressant effect by increased expression of 5-HT and BDNF receptors (rats, OE at 625, 1250 and 2500 μL/kg. Sub-chronic treatment).	[85]
2022 *Paeonia lactiflora* Pall.	palmitic acid (37.8%); linoleic acid (29.5%)	TST; FST; OFT; SPT	IP	FLU (3 mg/kg)	Antidepressant effect by downregulation of cortisol in brain tissues; reduction of corticosterone-induced neuronal damage; exerted anti-apoptotic effects on hippocampal neurons (mice, EO at 1 mg/kg and 3 mg/kg. Sub-chronic treatment).	[86]
2022 *Pogostemon cablin* Benth	patchoulol (60.66%)	TST	INH	FLU (20 mg/kg)	Effective antidepressant response based on immobility time in TST associated to increased level of dopamine in brain tissues (mice, EO at 60 g).	[36]
2022 *Schinus lentiscifolius* Marchand	δ-cadinene (21.0%); d-limonene (12.9%); 1-*epi*-cubenol (4.7%);	OFT; NFT; TST; FST; IT	IP	CPM (1.25 mg/kg)	Reduction of depressive symptoms observed by behavioral tests (Wistar rats, EO at 10 and 30 mg/kg. Acute treatment).	[87]
2022 *Tagetes minuta* L.	(*Z*)-tagetone (70.6%); (*Z*)-β-ocimene (11.1%); dihydrotagetone (4.7%)	OFT; TST; splash test	PO	---	Reversed the depressive-like behavior induced by stress (mice, EO at 10 and 50 mg/kg. Acute treatment).	[88]
2022 *Zanthoxylum bungeanum* Maxim.	linalool (19.2%); d-limonene (15.1%); linalyl acetate (13.8%)	CUMS; OFT; EPM; FST; TST	PO	SER (20 mg/kg)	Reduced CUMS-induced depression behavior by regulating the HPA axis and activating the PI3K/Akt signaling pathway (mice, EO at 50, 100 and 150 mg/kg. Sub-chronic treatment).	[84]

AM: Action Mechanism; PE: Pharmacological Effect; RA: Route of Administration; RD: Reference Drug; DZP: Diazepam; FLU: Fluxetine; DPM: Desipramine; RSP: Reserpine; SLM: Scopolamine; BPR: Buspirone; CPM: Clomipramine; IPM: Imipramine; SER: Sertraline hydrochloride; EPM: Elevated Plus-Maze Test; HBT: Hole Bboard Test; YMT: Y-Maze Task; RAMT: Radial Arm-Maze Test; TST: Tail Suspension Test; OFT: Open Field Test; FST: Forced Swimming Test; CUMS: Chronic Unpredictable Mild Stress; SPT: Sucrose Preference Teste; NSFT: Novelty-Suppressed Feeding Test; IT: Immobility Time; IP: Intraperitoneal; INH: Inhalation; PO: Oral Administration; ICV: Intracerebroventricular.

**Table 2:** Binding energy (kJ/mol) calculated by molecular docking of the main monoterpenes, sesquiterpenes and phenylpropanoids present in EO with antidepressant effects in the protein SERT and DAT.

Compound Class	Compounds	SERT	DS_norm-SERT_	DAT	DS_norm-DAT_
Neurotransmitter	serotonin	−99.70	−108.52	-	-
	dopamine	-	-	−77.07	−86.31
Control	fluoxetine	−108.28	−115.30	-	-
Monoterpenes	(*S*)-(+)-linalool	−79.01	−92.83	−72.35	−85.86
	(*R*)-(−)-linalool	−79.40	−90.32	−79.56	−92.76
	(+)-terpinen−4-ol	−66.70	−76.86	−52.25	−66.90
	(−)-terpinen−4-ol	−65.45	−76.00	−52.30	−66.92
	d-limonene	−68.29	−83.92	−58.68	−69.19
	l-limonene	−67.69	−83.45	−54.50	−67.63
	γ-terpinene	−67.07	−81.86	−56.62	−70.18
	(*R*)-(−)-α-phellandrene	−68.36	−84,08	−52.59	−65.65
	(*S*)-(+)-α-phellandrene	−67.00	−83.90	−51.37	−65.66
	(+)-α-pinene	−64.70	−49.97	−51.37	−65.05
	(−)-α-pinene	−64.40	−50.86	−52.21	−66.03
Sesquiterpenes	(−)-β-caryophyllene	−86.53	−96.20	−73.18	−58.55
	patchoulol	−47.36	−56.12	−66.05	−72.52
Phenylpropanoids	(*E*)-anethole	−72.53	−87.82	−55.91	−64.0
	(*E*)-cinnamaldehyde	−65.18	−82.77	−58.84	−72.76

## Data Availability

Data is contained within the article.

## Abbreviations

AM	Action Mechanism
BPR	Buspirone
COVID-19	Coronavirus disease 2019
CPM	Clomipramine
CUMS	Chronic Unpredictable Mild Stress
DPM	Desipramine
DZP	Diazepan
EPM	Elevated Plus-Maze Test
FST	Forced Swimming Test
FLU	Fluxetine
HBT	Hole Bboard Test
ICV	Intracerebroventricular
INH	Inhalation
IP	Intraperitoneal
IPM	Imipramine
IT	Immobility Time
NSFT	Novelty-Suppressed Feeding Test
OFT	Open Field Test
PE	Pharmacological Effect
PO	Oral Administration
RA	Route of Administration
RAMT	Radial Arm-Maze Test
RD	Reference Drug
RSP	Reserpine
SER	Sertraline hydrochloride
SLM	Scopolamine
SPT	Sucrose Preference Teste
TST	Tail Suspension Test
YMT	Y-Maze Task
